# Postoperative outcomes and stimulation responses for sectioned nerve roots during selective dorsal rhizotomy in cerebral palsy

**DOI:** 10.1007/s00701-024-06187-8

**Published:** 2024-07-30

**Authors:** Ziyad Makoshi, Monica Islam, Jennifer McKinney, Jeffrey Leonard

**Affiliations:** 1 Neurosciences Department, El Paso Children’s Hospital, El Paso, TX USA; 2 Department of Surgery, Paul L. Foster School of Medicine, Texas Tech University Health Sciences Center El Paso, El Paso, TX USA; 3 Division of Pediatric Neurology, Department of Pediatrics, The Ohio State College of Medicine, Nationwide Children’s Hospital, Columbus, OH USA; 4 Department of Pediatric Neurosurgery, Nationwide Children’s Hospital, Columbus, OH USA; 5 The Ohio State University Wexner Medical Center, The Ohio State University College of Medicine, Columbus, OH USA

**Keywords:** Selective dorsal rhizotomy, Cerebral palsy, Spasticity, Baclofen, Botulinum toxin

## Abstract

**Background:**

Cerebral palsy (CP) is the most cause of motor dysfunction in children. Selective dorsal rhizotomy (SDR) plays a major role in long term spasticity control. However, limited data exists on the effect of SDR on postoperative spasticity treatment requirements and supraspinal effects, and the stimulation responses of dorsal nerve roots in those with CP.

**Methods:**

The current study included the outcome for 35 individuals undergoing SDR for motor functional outcome, spasticity, baclofen dose changes, botulinum toxin injection frequency, and spasticity related orthopedic procedures. We also report on the stimulation responses in 112 individuals who underwent SDR at our institution.

**Results:**

There was a significant difference in gross motor function measures (GMFM)-66 scores at last follow up that remained present when considering only ambulatory children but not with non-ambulatory children. Ashworth scores were significantly decreased for both upper and lower extremities after SDR at all follow up points. There was a significant decrease in Baclofen dose and botulinum toxin injections requirements after SDR, but no significant difference in the need for orthopedic intervention. A total of 5502 dorsal nerve roots were tested showing a decrease in stimulation intensity and increase in grade on the right side and for descending lumbosacral levels.

**Conclusions:**

SDR improves gross motor scores during short term follow up but has additional benefits in decreasing baclofen dosing and botulinum toxin injections requirements after surgery. They stimulation responses of sectioned dorsal nerve roots adds to the limited available data and our understanding of the pathological changes that occur in CP.

**Supplementary Information:**

The online version contains supplementary material available at 10.1007/s00701-024-06187-8.

## Introduction

Cerebral palsy (CP) is the most common cause of motor dysfunction in children, with spasticity playing a prominent role in the lives of these individuals [11, 65]. The degree to which spasticity impacts an individual’s function can be assessed by the gross motor function measure (GMFM), originally developed to follow the effects of physical therapy in patients with CP [67], and commonly used to follow the natural history and effects of treatments. The 66-itemized version of the GMFM is a reliable tool to assess individuals with CP on five dimensions [68]. Based on the GMFM, the gross motor function classification system (GMFCS) was later developed based on the GMFM tasks into five levels, whereby those classified as levels I to III are predominately ambulatory and IV and V are not [57]. These measures serve to stratify individuals in quality of life surveys, treatment outcomes, and clinical research purposes.

Spasticity is a significant burden on individuals with CP and their caregivers [24]. In 1911, Foerster reported on early attempts to manage spasticity surgically with posterior spinal nerve root resection [17]. However, despite its benefits in tone reduction, there were significant postoperative complications, and its utilization remained limited to a small subset of surgeons until its revival by Gros in the 1960’s [23]. In the 1970’s, Fasano et al. reported on their experience with a functional approach to selective dorsal rhizotomy (SDR) using intraoperative stimulation and recording of electromyography (EMG) responses thus forming the basis for stimulation parameters used today [16]. The two modern approaches most commonly employed include the multi-level laminectomy SDR introduced by Peacock in 1987[62] and a refined limited laminectomy SDR at the level of the conus described by Park in 1993 [58]. Around the same time, other non-permanent options were also being sought for individuals with spasticity. Baclofen was introduced in the 1970’s as a potential treatment for spasticity in CP [8], but intolerance remained a major issue for some individuals. In the 1990’s, botulinum toxin injections into target muscles also showed benefit for treating spasticity [35]. These treatments, although reversable, are not without their cost and side effect profile.

The effect of SDR on the need for ongoing spasticity therapies remains limited. The current study aimed to assess multiple pre- and postoperative parameters of individuals with CP treated with SDR at our institution. We aimed to report not only functional changes after the procedure, but also its effect on requiring other spasticity treatments. In addition, we report on the intraoperative stimulation responses of sectioned dorsal nerve roots in individuals with CP undergoing SDR to add to the limited published electrophysiological data.

## Methods and materials

### Study design

This is a retrospective review of 117 patients who underwent SDR by the senior author (J.R.L) between January 2015 and August 2021 at Nationwide Children’s Hospital. Some patients were referred from out of city/state/country and long term follow up was performed elsewhere. The study had two objectives, analysis of participant outcome data with follow up at least 10 months postoperatively (available for 35 patients) and analysis of the intraoperative electrophysiological data (available for 112 patients). The outcome group baseline data included age at surgery, gender, spasticity type, presence of dystonia, preoperative Ashworth grade [2], GMFCS level, GMFM-66 score and percentile [24]. Outcome measures included postoperative Ashworth grade, GMFCS level, GMFM-66 scores, baclofen use and dose, botulinum toxin injections, and spasticity-related orthopedic procedures. The Ashworth scale is used more commonly by our physiatrists and was therefore used for analysis. Electrophysiological baseline data included age, gender, GMFCS level, and spasticity type. The Intuitional Review Board at Nationwide Children’s Hospital approved the current study.

Individuals with CP related spasticity referred to our center are candidates for SDR if: spasticity affects their quality of life, they are ambulatory with or without assistance, they are able to participate in rigorous postoperative physiotherapy, dystonia is not a major component of their presentation, they have spastic diplegia, and other causes of spasticity are ruled out by clinical assessment and cranial neuroimaging. Non-ambulatory individuals are considered for SDR as a palliative option on a case-by-case basis after discussion with the primary caregivers if decreasing spasticity is likely to have a positive impact on quality of life and individual care. Both types of candidates were included in this cohort.

All ambulatory individuals were enrolled in postoperative physiotherapy on day 4 – 5, non-ambulatory patients are discharged home when ready. Postoperative physiotherapy generally includes inpatient rehabilitation for approximately 2 weeks, followed by outpatient sessions approximately 4 – 5 times per week for six months, then 2 – 3 times per week for six to twelve months modified based on the individual needs and progress. All individuals were assessed by a trained physiotherapist and/or physiatrist on admission to rehabilitation. Based on follow up times among participants with GMFM-66 assessments, this was divided into short term (0 – 7 months), intermediate term (8 – 19 months), and long term follow up (≥ 19 months). Based on available follow up times among patients with Ashworth assessments, the timeframe was grouped into four time periods: 0 – 3 months, 4 – 9 months, 10 – 19 months, and > 19 months to reflect changes over time.

### Surgical technique

**Fig. 1:**
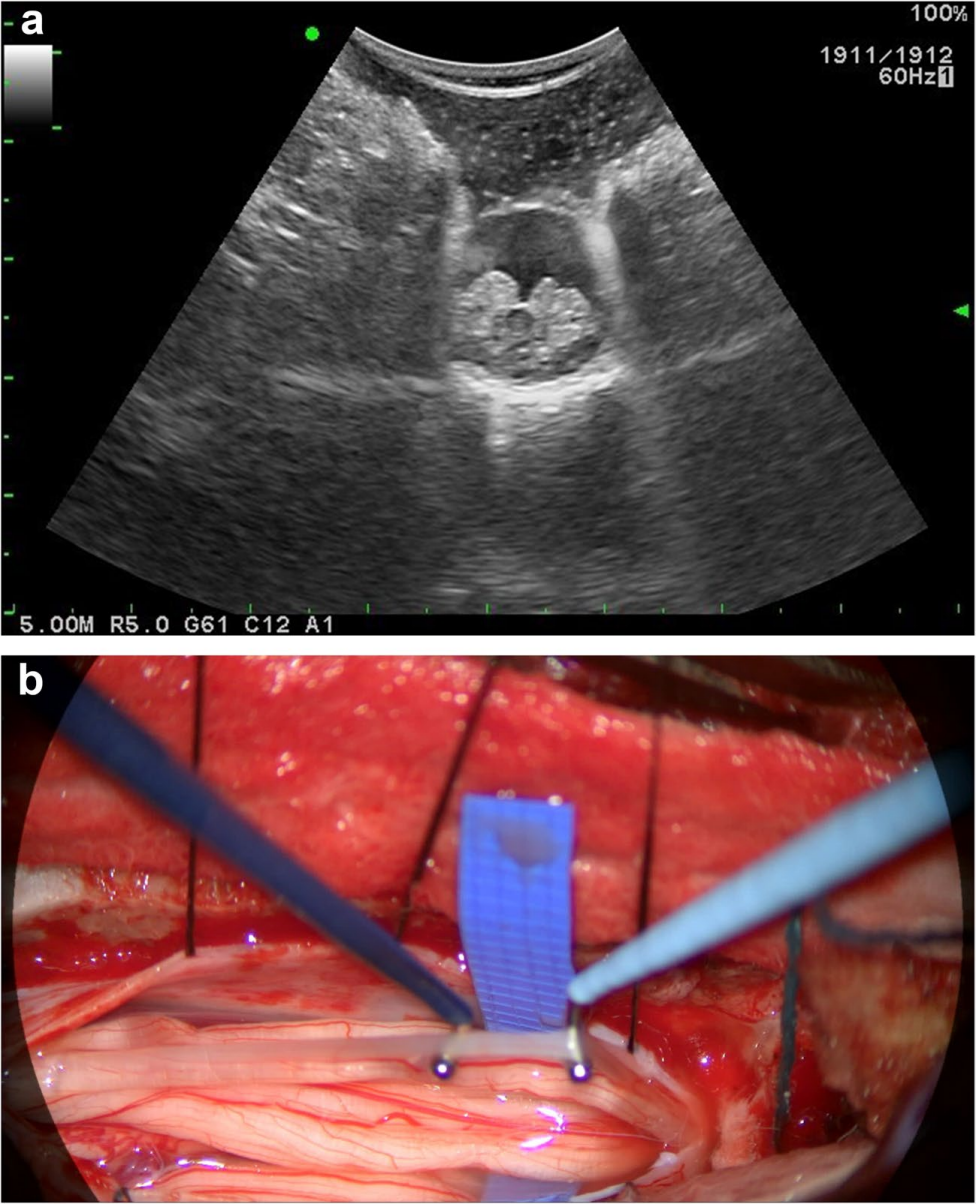
(**a**) Intraoperative ultrasound prior to laminectomy to determine level of transition from conus (central dark grey) to nerve roots (surrounding light grey structures). (**b**) Intraoperative microscopic image of isolated right sided dorsal nerve roots before starting rhizotomy, terminal end of conus appreciated (superior [left] midline structure)

Surgical approach is similar to that described by Park and Johnston [61]. The patients are positioned prone with EMG monitoring of the lower extremity and the anal sphincter. No botulinum toxin injections are given within 6 months of surgery for reliable monitoring. The incision is made along the midline over the L1 spinous process and the L1-L2 interspinous space is visualized with the intraoperative ultrasound to identify the conus and cauda equina transition level (Fig. 1). Once the transition level is confirmed, a single level laminectomy is performed. The dura is opened and the dorsal nerve roots on the more severely affected side (if one is present on exam or expressed by the parents) are targeted first in the event surgery cannot be completed due to any unforeseeable complications. The roots are isolated with a silicone strip without including the smaller midline sacral nerve roots (S3-S5). Each nerve root is then isolated and stimulated with rhizotomy probes to identify the level. Depending on size, the root is then divided into 3 – 5 rootlets and sectioned rootlets are stimulated for threshold and grade. We aim to cut approximately two-thirds of each dorsal nerve root and therefore, non-sectioned rootlets were not routinely stimulated or recorded. If a nerve root produces compound muscle action potentials with stimulation < 0.5 mA then mechanical stimulation of the nerve root is performed to help define whether this is a ventral nerve root which should be spared. Any nerve root with an isolated or predominant sphincter response is spared. After sectioning is completed on one side, the L1 nerve root is identified at its foraminal exit, and 50% is cut without stimulation. The same procedure is then carried out on the contralateral side. The dura is closed in watertight fashion, and an epidural catheter is then placed. The muscle, fascia and skin are then closed in layers. Post-operative pain control includes epidural analgesia for three days and as needed oral and intravenous analgesia; this postoperative pain protocol has been described previously [27].

All SDR procedures at our institution are done with neurophysiologic intraoperative monitoring (NIOM). NIOM utilizes free run and stimulated EMG recordings of muscles representing innervation from the L2-S4 spinal levels. These consist of the bilateral adductor longus (L2-L4), vastus lateralis (L2-L4), tibialis anterior (L4-L5), short head of the biceps femoris (L5-S1), medial gastrocnemius (S1-S2), abductor hallucis brevis (S2-S3), and external anal sphincter (S3-S4). The spinal level for each root is determined based on what muscles demonstrate compound muscle potentials with 1 Hz stimulation. Intensity threshold for each rootlet is determined by the lowest level of stimulation needed to produce a consistent compound muscle potential. Each rootlet then undergoes tetanic 50 Hz stimulation to allow for grading of the EMG response. The Philips and Park grading scheme defines a 0–4 scale to grade responses with Grade 4 being the most severe [63]. Neuromonitoring data recorded for each nerve root included laterality, spinal level based on nerve root stimulation at 1 Hz, rootlet threshold intensity (mA) at 1 Hz, rootlet grade at 50 Hz, and number of rootlets cut. The spinal level for each root was designated as lumbar (L2-L5 spinal levels), lumbosacral (spanning lumbar and sacral spinal levels), or sacral (S1-S4 spinal levels).

### Statistical analysis

Data analysis was performed with IBM SPSS Statistics® v29 (IBM Corp, Armonk, NY, USA). Descriptive data was presented as mean, standard error (SE) and SE of the mean, and percentage. For continuous data, a paired-sample *t*-test was used to compare preoperative and postoperative results. For categorical data, Chi-squared test or Fisher’s exact test was used when appropriate, and odds ratio (OR). For continuous independent variables, independent-sample *t*-test was used to assess differences in means. Pearson correlation coefficient was used to assess the association between continuous variables, while Spearman’s Rank-Order Correlation was used to assess the association between continuous and ordinal data. Changes over time were plotted using one-way repeated measures analysis of variance (ANOVA) for continuous variables and Wilcoxon Signed-Rank Test for categorical variables, and McNemar test for nominal data comparing pre- and postoperative baclofen use, spasticity related orthopedic surgery and botulinum toxin injections. The Mann–Whitney U test was used to compare differences between groups in pre- and postoperative dichotomous outcome measures (baclofen use, orthopedic surgery, and lower extremity botulinum toxin injection) for ordinal variables (grade at 50 Hz). One-way ANOVA was used to assess differences in electrophysiological data based on patient variables, with Tukey honestly significant difference (HSD) post hoc analysis used when a significance difference was encountered. Linear regression was used to assess relationship between intraoperative electrophysiological parameters and age and percentage of rootlets cut. Standard error (SE) or standard deviation (SD) and 95% confidence intervals (CI) were reported were possible and confirmed with bias-corrected and accelerated (BCa) bootstrap confidence intervals (1000 samples).

To ensure equal weighting of NIOM data with the variation in the number of rootlets per participant, comparisons with other variables were performed against the average stimulation and grade per participant and per participant side and level. For subgroup analysis, GMFCS levels were divided into ambulatory (GMFCS levels I – III) and non-ambulatory (GMFCS levels IV and V). The Ashworth scale was divided into upper and lower extremity, and the lower extremity was further divided into lumbar (L1-L5): hip flexion and adduction and knee extension; lumbosacral (L5-S1): hip extension and abduction, knee flexion, and ankle dorsiflexion; sacral (S1-S3): ankle plantarflexion; in addition to laterality.

## Results

### Outcome group characteristics for 35 patients

A total of 35 patients were available. Mean age was 7.11 ± 3.73 years (range 3 – 18), and 15 (42.9%) patients were female. The most common spasticity subtype was diplegic spastic CP in 18 participants (51.4%). Twenty-eight (96.65%) participants had periventricular white matter changes on 29 available MRI brain images, and 8/32 (25%) had ventriculoperitoneal shunts. Patient characteristics are shown in Table 1. Follow up beyond 19 months was available for 31/35 patients (88.57%), and mean last available follow up was 28.2 ± 12.6 months (range 10 – 62 months).

**Table 1:** Participant characteristics undergoing selective dorsal rhizotomy with outcome data (*n* = 35)

*Patient Characteristics*	*N*	*%*
Age (mean ± SD)	7.11	± 3.73
< 10 years	28	80
≥ 10 years	7	20
Gender		
Male	20	57.1
Female	15	42.9
GMFCS Level		
I	11	31.4
II	7	20
III	9	25.7
IV	5	14.3
V	3	8.6
Spasticity Type		
Diplegia	18	51.4
Hemiplegia	4	11.4
Tetraplegia	5	14.3
Quadriplegia	8	22.9
Dystonia		
Present	2	5.7
Not present	33	94.3
Ventriculoperitoneal Shunt		
Yes	8	22.86
No	24	68.57
Not available	3	8.57
MRI findings		
No abnormality	1	2.86
Periventricular leukomalacia	27	77.14
Ventriculomegaly/Periventricular gliosis	1	2.86
Not available	6	17.14

Abbreviations: *SD* standard deviation, *GMFCS* gross motor function classification system, *MRI* magnetic resonance imaging

### GMFCS, GMFM-66, and Ashworth scores

There was no statistically significant change in GMFCS level at any follow up point (for change in level at ≥ 19 m, Z = -0.816, p = 0.414). GMFM-66 score before SDR and ≥ 19 months follow up were strongly and positively correlated (r = 0.84, p < 0.001). When considering only ambulatory patients, a paired-samples *t*-test showed a significant difference between preoperative and ≥ 19 m scores;(t16) = -2.75, p = 0.014). On average, ≥ 19 month follow up scores were 5.6 points higher than preoperative scores (95% CI 9.96, -1.28). There was no significant change between the preoperative score and any earlier time points, and no significant change for percentile before and after surgery (supplementary Table 1).

**Fig. 2:**
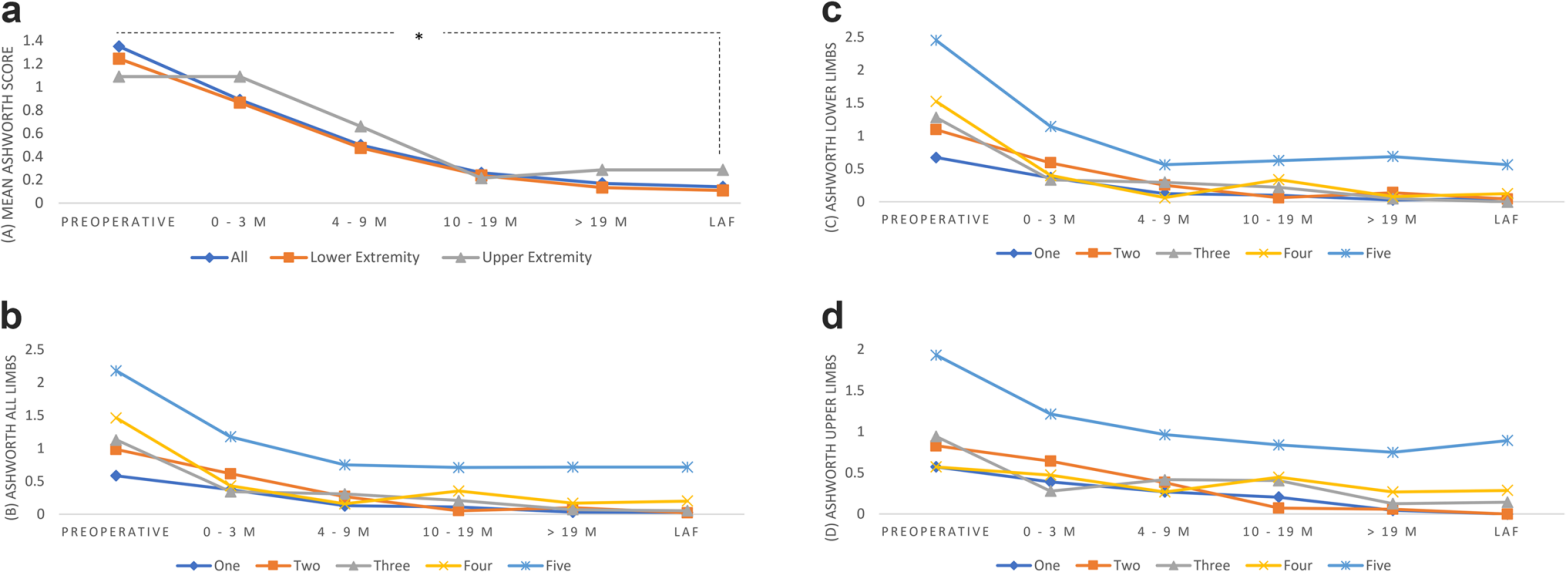
Mean Ashworth scores preoperatively and postoperatively based on (**a**) limbs and (B-D) GMFCS level, (**b**) all limbs, (**c**) lower extremity, (**d**) upper extremity. Asterisk indicates statistically significant difference obtained at p < 0.001

A total of 1050 limbs were assessed for spasticity, lower extremity joints included 560 (53.3%) limbs and upper extremity were 490 (46.7%) limbs, equally divided on the right and left. A paired-samples *t*-test was conducted to compare Ashworth scores before and after SDR. The results indicate a significant difference between Ashworth scores before SDR (M = 1.06; SD = 1.085) and after SDR at follow up > 19 months (M = 0.12; SD = 0.47); [t(625) = -23.213, p < 0.001]. A significant decrease was seen for all follow up time points and when considering upper and lower extremities separately (Fig. 2, supplementary Table 2).

### Outcome measures

Twenty-seven (77.1%) patients were receiving preoperative botulinum toxin injections to the lower extremities. There was no significant difference between ambulatory and non-ambulatory patients to receive lower extremity botulinum toxin injections (p = 0.648), but postoperatively non-ambulatory patients were 21 times more likely to continue to receive injections compared to ambulatory patients (OR 21.15, 95% CI 0.9 – 495.9, p = 0.047). There was a significant difference in the proportion of botulinum toxin injections pre- and postoperatively overall (p < 0.001) and for ambulatory patients (p < 0.001), but was not significant when considering only non-ambulatory patients (p = 0.063).

Mean recorded follow up for postoperative baclofen use was 23.75 ± 18.67 months (range 2 – 56 months). Non-ambulatory patients were 14 times more likely to be taking baclofen preoperatively (OR 14.09, 95% CI 1.486 – 125, p = 0.013 using Fisher’s exact test), but were not significantly different when considering dose (mg/24H) despite higher doses in non-ambulatory patients (M = 9.096, SD = 24.357) than ambulatory patients (M = 17.563, SD = 11.121); (t(32) = -0.945, p = 0.351). There was a significant difference in the proportion of those receiving baclofen pre- and postoperatively overall (p = 0.003) and for ambulatory patients (p = 0.021), but was not significant when considering only non-ambulatory patients (p = 0.25). Mean baclofen dose differed before and after SDR (F(1262.485, 242.644) = 5.203, p = 0.029). Baclofen dose was decreased from preoperatively to 12 months postoperatively (mean difference = 8.618 mg/24H, 95% CI 0.931 – 16.304, p = 0.029). This significance was lost in the subgroup analysis based on ambulatory status Table 2.

**Table 2:** Preoperative and postoperative comparisons for spasticity interventions (*n* = 35): baclofen, botulinum toxin, and Orthopedic surgery

Preop GMFCS Level	Preop Baclofen				Postop Baclofen at 12 months				Preop Botox (N, %)						Postop Botox (N, %)						Preop Ortho Srx		Postop Ortho Srx	
	N	%	µDose (mg/24H)	SD	N	%	µDose (mg/24H)	SD	Any	%	LE	%	UE	%	Any	%	LE	%	UE	%	N	%	N	%
Overall	16	45.7	11.1	22.1	5	14.3	2.5	7	28	80	27	77.1	4	11.4	5	14.3	2	5.7	5	14.7	6	17.1	10	28.6
p-value^*^					**0.003**		**0.029**								** < 0.001**		** < 0.001**		**1**		0.344			
A (n = 27)	9	33.3	9.1	24.4	1	3.7	0.2	1	21	77.8	20	74.1	3	11.1	3	11.1	0	0	3	11.5	3	11.1	6	22.2
1 (n = 11)	3	27.3	6.36	11.4	1	9.1	0.45	1.5	9	81.8	9	81.8	1	9.1	1	9.1	0	0	1	9.1	1	9.1	1	9.1
2 (n = 7)	2	28.6	17.9	45.1	0	0	0	-	6	85.7	5	71.4	1	14.3	1	14.3	0	0	1	14.3	1	14.3	3	42.9
3 (n = 9)	4	44.4	5.2	9.2	0	0	0	-	6	66.7	6	66.7	1	11.1	1	11.1	0	0	1	12.5	1	11.1	2	22.2
p-value^*^					**0.021**		0.075								** < 0.001**		** < 0.001**		1		0.375			
NA (n = 8)	7	87.5	17.6	11.1	4	50	9.9	12.1	7	87.5	7	87.5	1	12.5	2	25	2	25	2	25	3	37.5	4	50
4 (n = 5)	5	100	17.3	9.5	3	60	9.8	10.3	5	100	5	100	1	20	2	40	2	40	2	40	1	20	3	60
5 (n = 3)	2	66.7	18	15.9	1	33.3	10	17.3	2	66.7	2	66.7	0	0	0	0	0	0	0	0	2	66.7	1	33.3
p-value^*^					0.25		0.12								0.063		0.063		1		1			

^*^McNemar test and ANOVA repeated measures (baclofen dose) used as appropriate, all tests are two-sided

Percentages are based on total numbers for each subgroup

Abbreviations: *GMFCS* gross motor function classification system, *A* ambulatory, *NA* non-ambulatory, *Preop* preoperative, *Postop* postoperative, *Ortho* orthopedic, *Srx* surgery, *N* number, *µ* mean, *mg* milligrams, *SD* standard deviation, *LE* lower extremities, *UE* upper extremities

There was no significant difference between the rates of preoperative and postoperative orthopedic surgeries for spasticity (p = 0.344). Non-ambulatory participants had a higher rate of postoperative orthopedic surgeries compared to ambulatory patients but the difference was not significant (22.2 vs 50%, p = 0.186). Results are shown in Table 4.

### Clinical characteristics and sectioned nerve root stimulation responses for 112 patients

**Fig. 3:**
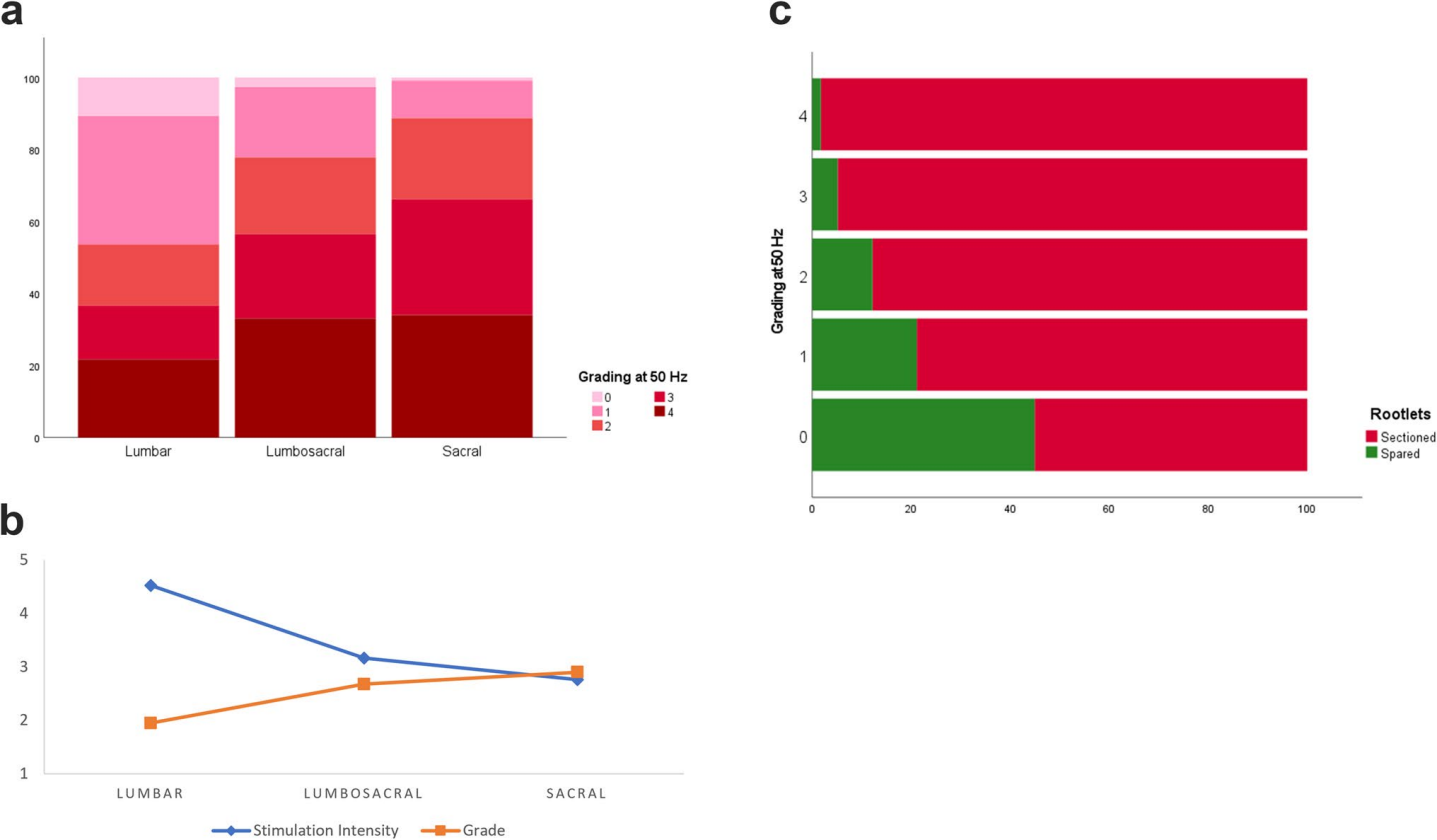
(**a**) Grading distribution (percent) based on spinal level. (**b**) Mean stimulation intensity and grading at 50 Hz based on spinal level. (**c**) Stacked bar chart for rootlets sectioned (percent) based on grade

A total of 5502 sectioned nerve rootlets for 112 patients were included in the final analysis. Mean age was 8.34 ± 5.43 years (range 3 – 28), and 62 were males (55.36%). CP type was available for 105 patients of which the majority were spasticity without dystonia (n = 100) and 5 were mixed. The average number or nerve roots per patient was 48 ± 10.84 (mode 47, min 20 and max 84). Nerve roots were divided on average to 3.38 ± 0.848 rootlets (range 1 to 7, mode 3), and 65.6% ± 8.04% on average was cut. Stimulation responses and patient characteristics are shown in Table 3. Differences and predictors between variables and electrophysiological data are shown in Table 4. There was a significant difference between sides (left and right) for stimulation intensity (left side mean increase of 1.59 mA) and grading (left side mean decrease of 0.716). The percentage of rootlets cut was significantly predicted by stimulation intensity (p < 0.001) and grading (p < 0.001). The fitted regression model was: percentage of rootlets cut = 65.85%—0.067 (stimulation intensity in mA), and 64.61% + 0.407 (grading at 50 Hz). A one-way ANOVA showed a significant difference between spinal levels in stimulation intensity (p < 0.001) and grading (p < 0.001); a Tukey’s HSD Test for multiple comparisons was performed and results are shown in Table 5. Figure 3 graphically shows this relationship.

**Table 3:** Nerve root characteristics among 112 participants who underwent selective dorsal rhizotomy

Nerve Roots		*N*	%	Stimulation intensity (mA)		Grading at 50 Hz		Rootlets Cut (%)	
				Mean	SD	Mean	SD	Mean	SD
Overall		5502	100	3.711	4.988	2.41	1.287	65.6	8.04
Age	< 10 years	77	68.75	3.81	5.154	2.44	1.28	65.83	8.39
	≥ 10 years	35	31.25	3.506	4.618	2.36	1.3	65.1	7.2
Gender	Male	62	55.36	3.235	4.77	2.38	1.287	65.88	8.67
	Female	50	44.64	4.31	5.189	2.45	1.286	65.23	7.11
Level	Lumbar	2616	47.5	4.655	5.824	2.01	1.34	65.4	8.5
	Lumbosacral	1344	24.4	3.07	3.935	2.65	1.204	65.38	7.8
	Sacral	1542	28	2.67	3.848	2.88	1.025	66.11	7.39
Laterality	Left	2763	50.7	3.549	4.708	2.52	1.287	65.91	8.17
	Right	2683	49.3	3.887	5.289	2.31	1.285	65.27	7.89
GMFCS	I	26	23.6	4.327	5.115	2.54	1.261	64	8.46
	II	26	23.6	4.913	6.977	2.28	1.24	65.39	5.49
	III	33	30	2.78	3.128	2.4	1.317	65.74	8.51
	IV	17	15.5	2.683	2.926	2.39	1.3	66.17	6.6
	V	8	7.3	4.577	4.855	2.62	1.29	67.78	5.58
Spasticity	Diplegia	63	60	3.559	5.011	2.4	1.264	65.31	8.03
	Hemiplegia	12	11.4	3.839	4.373	2.61	1.333	67.07	8.98
	Triplegia	9	8.6	5.056	6.955	2.19	1.372	65.56	10.13
	Quadriplegia	21	20	3.497	4.536	2.39	1.301	65.69	7.55

Missing data: Laterality = 56, GMFCS = 2, Spasticity = 7

Abbreviations: *mA* milliamperes, *Hz* hertz; *SD* standard deviation, *GMFCS* gross motor function classification system

**Table 4:** Differences and predictors for stimulation intensity and grading

Variable	Stimulation Intensity (mA)		Grading at 50 Hz	
*One-way ANOVA for differences between groups*				
Gender	F(1, 112) = 2.485	p = 0.118	F(1, 112) = 0.272	p = 0.603
Spinal Level	F(2, 326) = 8.08	**p < 0.001**	F(2, 326) = 49.906	**p < 0.001**
Laterality	F(2, 220) = 11.294	**p < 0.001**	F(1,220) = 56.263	**p < 0.001**
Gender	F(1, 110) = 0.364	p = 0.547	F(1, 110) = 0.379	p = 0.54
GMFCS	F(4,105) = 1.345	p = 0.258	F(4, 105) = 1.24	p = 0.298
Spasticity	F(3, 103) = 0.066	p = 0.978	F(3, 103) = 1.322	p = 0.271
*Linear regression for possible predictors*				
Age	R^2^ = 0.001, F(1, 110) = 0.153	p = 0.696	R^2^ = 0.003, F(1, 110) = 0.313	p = 0.577
GMFM-66	R^2^ = 0.027, F(1, 57) = 1.593	p = 0.212	R^2^ = 0.004, F(1, 57) = 0.2	p = 0.656
% Rootlets cut	R^2^ = 0.002, F(1, 5384) = 8.794	**p = 0.003**	R^2^ = 0.004, F(1, 5379) = 23.058	**p < 0.001**

One-way ANOVA reported as: F(df between groups, df within groups) = F-value, p-value

Linear regression reported as: R squared value, F(df regression, df residual) = F-value, p-value

Abbreviations: *mA* milliamperes, *Hz* hertz, *ANOVA* analysis of variance, *GMFCS* gross motor function classification system, *GMFM* gross motor function measure

**Table 5:** Tukey post hoc analysis for stimulation intensity and grading based on spinal level

Spinal Level		Stimulation Intensity (mA)					Grading at 50 Hz				
		MD	SE	Sig	95% CI		MD	SE	Sig	95% CI	
Lumbar	LS	1.360	0.462	**0.01**	0.271	2.448	-0.728	0.100	** < 0.001**	-0.962	-0.493
Lumbar	Sacral	1.759	0.459	** < 0.001**	0.678	2.840	-0.947	0.099	** < 0.001**	-1.181	-0.713
LS	Sacral	0.399	0.464	0.666	-0.694	1.492	-0.220	0.100	0.074	-0.456	0.016

Abbreviations: *mA* milliamperes, *Hz* hertz, *MD* mean difference, *SE* standard error, *Sig* significance, *CI* confidence interval, *LS* lumbosacral

## Discussion

### Functional outcomes after SDR

This study considered both GMFCS level and GMFM-66 changes after SDR. GMFCS level did not significantly change, but a difference was found in GMFM for ambulatory individuals. The natural history of motor function over time suggests that major gains in ambulatory children with CP occur up to the age of 6 to 7 years and then plateaus [4, 26, 66]. This would not explain earlier or later gains seen at various age groups in our study. One factor that may play a role is the wide variation in reported outcomes between and among GMFCS levels, and that motor gains can occur earlier and to a much lesser degree in non-ambulatory individuals [66, 71]. Hanna et al. found that some functional decline may occur in GMFCS levels IV and V after reaching their early childhood peak as they enter into adolescence and early adulthood, and the decline was most prominent among those with GMFCS level III [25]. This decline has not been demonstrated by other reports [71]. McLaughlin et al. found the greatest gain in GMFM was among non-ambulatory children although this did not reach significance [42]. Clearly, variation exist in the outcome for older children and this may be secondary to other factors, as shown by Bartlett et al. where range of motion, scoliosis, and pain correlated with a decline in adolescents with GMFCS III, IV, and V [3].

GMFCS level III formed nearly a third of our cohort. A systematic review of walking performance from childhood to adulthood found that GMFCS level III carries the highest variability to improve, remain stable, or decline with time, while those above or below this level largely remained unchanged [10]. Two of three randomized control trials on SDR involving ambulatory individuals found a significant change in GMFM scores at 9 to 12 months [42, 73, 83], and a meta-analysis of these trials similarly found significant improvement when data was pooled [43]. Long term significant improvement in GMFM scores for ambulatory children has been reported 5 to 10 years after SDR [6, 15, 31, 76], but not for non-ambulatory children [15]. Tedroff et al. reported a gradual decline at ten years compared to earlier gains [76, 77], but remained a significant improvement from baseline at ten years [76], and was no longer significant by 17 years [77]. Their study only included 18 participants of which 7 were non-ambulatory. A larger cohort of 100 individuals reported by Park et al. included childhood SDR from all GMFCS levels with sustained or even improved function at 20 to 28 years [59]. Unfortunately, neither Tedroff et al. or Park et al. stratified their analysis based on ambulatory status to provide further insight into long term outcomes in each of these groups. These studies show the wide variations both in the natural history of CP and in those undergoing SDR. However, similar to our findings, early and sustained short term gains occur for ambulatory children undergoing SDR, but further subgroup analysis including long-term functional outcome is needed to better predict who will continue to benefit and who may not.

### Limb spasticity and SDR

We found a significant decrease in spasticity in all limbs at last follow up after SDR, this included the upper and lower extremities. This is in keeping with reported SDR benefits in decreasing spasticity in the lower extremities in three randomized controlled trials at 9 to 12 months [43], and these effects were maintained long term [1, 39, 77]. Its effect on upper extremity tone and function however is less discussed. Early studies on changes in tone in the upper extremities after SDR in ambulatory children were not consistent [7, 62]. Ambulatory children are less likely to have spasticity in the upper extremity and therefore detection of a meaningful change may be difficult especially in smaller cohorts. In studies including individuals with spastic quadriplegia both tone and function of the upper extremity appear to improve with SDR [21, 46]. Other studies have also shown improvement in upper extremity function that continues several years after SDR [55, 72]. These changes may be due to suprasegmental effects, with a small case series suggesting changes at the cortical level [55], including cognitive gains compared to controls in a slightly larger cohort [12]. The evidence and quality of current available studies is not enough to make conclusive remarks, but strongly suggests that SDR likely has effects well above the level of operation and is an important aspect to be included in future research on SDR outcomes as this effects the indications for utilizing the procedure.

### Baclofen use and botulinum toxin injections after SDR

Our study found a significant post-operative decrease in the use of baclofen and botulinum toxin injections in ambulatory individuals, and this was sustained over the follow-up period. Additionally, there was a decrease in utilization in the non-ambulatory group, but this did not reach significance. Although the majority of SDR studies focus on motor function outcomes after SDR, an important and much less reported outcome is the change in baclofen use and botulinum toxin injections. A significant decrease in postoperative muscle relaxant use among SDR participants at one year follow up has been reported [79]. Of those that report on post-SDR anti-spasmodic medication or botulinum toxin injections, this varies from 0 to 38% and 12.5 to 53%, respectively, but were not compared to preoperative rates [15, 30, 60, 77].

The rate of adverse events reported from botulinum toxin injections ranges from 3.6 to 23.2% [36, 47, 52, 56]. While in one study of family reported adverse events, 95 events were reported in 45 individuals [5]. These rates may also be underestimated [75]. Ambulatory children may not have significant clinical benefit between physiotherapy alone compared to physiotherapy and botulinum toxin injections, with higher costs for the latter [69]. In a population-based study that included children enrolled in Medicaid, those with CP had average annual costs 15 times higher than average, of which pharmacy costs accounted for 11% and overall oral baclofen use was reported in 13.5% [64]. Side effects have been reported in 20—40%[22, 41, 44] and fatigue, lethargy, or drowsiness in more than a third of individuals [13]. These are important factors to consider for individuals and their families living with CP where both cost and risk of side effects over time may play a large role in their quality of life, however, dedicated studies are lacking and our findings further support the benefit of SDR in the ongoing need for these treatments.

### Orthopedic interventions after SDR

We did not find a significant difference in the rate of orthopedic procedures before and after SDR (17 vs 29%, respectively), although rates were higher in non-ambulatory participants (22 vs 50%). Prior orthopedic surgery was not found to be associated with “poor” outcome after SDR [34]. Comparative studies show the rate of orthopedic surgery after the first year of SDR may be lower than those receiving intrathecal baclofen therapy (19 vs 41%, respectively) [32], however this is limited by the short follow up as other studies found no orthopedic surgery done within the first year after SDR but reached 18% by five years[45] and 84% by 10 years [51]. Post-SDR orthopedic procedure rates at approximately five years varies at 18% [45], 24% [9], 42% [38, 48], and 66% [50]. Longer follow up studies beyond 15 years reported even higher rates of 28% [15], 57% [59], and 74% [30]. Age at SDR may play a role, Chicoine et al. reported a significantly higher rate of any orthopedic procedure (before or after SDR) in those 5 years and older compared to those younger than 5 years (45 vs 22%, respectively), but similar post-SDR rates (both 19%) [9]. This age related difference was also found by O’Brien et al. but their analysis included only post-SDR rates and did not reach significance [50]. This may be less related to the SDR procedure itself, but to the age and functional status of participants included in the SDR study. Individuals with CP undergoing orthopedic surgeries at a younger age may be more likely to require further surgeries in the future [49]. In a cohort of independent versus assisted walkers, the rate of post-SDR orthopedic surgery was significantly higher in the latter (24 vs 51%, respectively) [51]. Therefore, the effect of SDR on the rate of orthopedic surgery is likely more complex than can be answered with the current state of evidence, with age and functional status playing a significant role, and larger long term data is needed.

### Sectioned dorsal nerve roots stimulation responses in SDR

In our cohort, the left side had lower stimulation intensity thresholds and higher grades compared to the right side. Stimulation intensity thresholds were lower at lumbar levels compared to sacral levels, and grade also increased from lumbar to sacral levels. There was no association found with age, gender, or GMFCS level. This pattern was also reported by De Vloo et al. in a cohort of 145 participants [78], and similar findings regarding increasing grade in lower levels have been reported by others [19, 81, 82]. We divide spinal levels into lumbar, lumbosacral and sacral rather than individual spinal levels based on our experiences and that of others that muscle response will often occur with stimulation of more than one spinal level [20, 63, 70]. We found a significant asymmetry in grading, higher on the left, which was also reported by Wolter et al. [82] but not found by others [78]. It is difficult to ascertain whether these findings are due to a higher proportion of participants with left more than right spasticity being captured in any one study resulting in a type 1 error, if addressing one side effects the response on the contralateral side which was not controlled for in our study, or whether a true asymmetry in CP individuals exists and further investigation into this distribution is needed.

The use of electrophysiology during SDR has been debated. Arguments to forgo neuromonitoring include shorter operative time [74], similar postoperative outcomes [37, 74], and potentially non-reproducible results with repeat stimulation in a small cohort [80]. One study provided a mathematical probability, stating that even if 50 – 75% of rootlets were sectioned at random this would include enough abnormal rootlets to achieve clinical benefit. It is the senior author’s practice to always cut approximately two-thirds of the dorsal nerve roots. A meta-analysis of three randomized controlled trials found a significant inverse relationship between changes in GMFM scores and the percent of dorsal nerve roots sectioned [43]. This was also reflected in other studies comparing the rate of nerve root sectioning and spasticity outcome [28, 72]. We include L1 and S2 in our rhizotomy procedure to avoid residual spasticity at the hip adductors and ankles respectively as has been shown by some authors [33]. However, the benefit of neuromonitoring is beyond simply spasticity outcomes. Bladder and bowel innervation can receive contributions from S1 and S2 [14, 29, 53]. Overlap in stimulation above this level triggering anal sphincter response has also been put forth by some authors as an unreliable use of NIOM [54]. Nerve roots with more abnormal responses have more degenerative changes on histology further supporting the use of electrophysiological sectioning in SDR [18]. Additionally, monitoring has been used for more tailored approaches by some authors [19, 84]. We have also found that monitoring helps guide the surgeon performing single level SDR when anatomy is distorted to ensure adequate representation of all levels and avoiding ventral nerve roots with lower stimulation thresholds as has been reported by others [40]. Therefore, it is our practice to continue to use neuromonitoring for these cases in addition to the neurophysiological data this adds to understanding the change in neuronal circuity in CP individuals with spasticity.

### Limitations

The current study has several limitations. Including its retrospective nature, the smaller number of patients included in the outcome measures and length of follow up. The effect of SDR on upper limb spasticity is reported but we did not assess change in function or quality of life as it pertains to this improvement and believe this an important aspect to include in future studies. Absolute baclofen dose was used for analysis and not weight based dosing, however, as these were used to compare each participant’s pre- and postoperative change, we believe the results accurately reflect any changes. We did not include the type of orthopedic procedures done for spasticity which may provide frequencies and trends. Spared nerve rootlets were not included in the analysis which would add further information about the neurophysiological characteristics of these nerves; we sectioned the first 2/3rd if they were a grade 3 or 4 and only stimulated further if a grade 0 – 2 was encountered first. We believe this achieves satisfactory results and decreases the time under general anesthesia with shorter surgery times.

## Conclusions

SDR improves GMFM-66 scores at short term follow up in GMFCS I to III levels. SDR also significantly decreases the need for postoperative botulinum injections and baclofen use. Lower stimulation threshold and higher grades were seen on the left side and with descending lumbosacral levels. The stimulation responses of sectioned nerve roots adds to limited available information regarding dorsal nerve root characteristics in patients undergoing SDR. These parameters should be included in future reports on SDR beyond the gross motor function as they also impact quality of life and our knowledge of the neurophysiology of the disease.

## Supplementary Information

Below is the link to the electronic supplementary material.

Supplementary file1 (DOCX 45 KB)

## Data Availability

Data is available at request and per our institutional guidelines for data sharing.

## Abbreviations

CP: Cerebral palsy
GMFM: Gross motor function measure
GMFCS: Gross motor function classification system
SDR: Selective dorsal rhizotomy
EMG: Electromyography
NIOM: Neurophysiologic intraoperative monitoring
ANOVA: One-way repeated measures analysis of variance
SE: Standard error
SD: Standard deviation
CI: Confidence intervals
BCa: Bias-corrected and accelerated
OR: Odds ratio
mA: Milliamperes
Hz: Hertz
