# Supplementary material for: Postoperative outcomes and stimulation responses for sectioned nerve roots during selective dorsal rhizotomy in cerebral palsy
Source: Acta Neurochir (Wien). 2024 Jul 30;166(1):308. doi: 10.1007/s00701-024-06187-8 (PMC11289343; doi:10.1007/s00701-024-06187-8)
Supplement: Supplementary file 1 — Supplementary file1 (DOCX 45 KB) [file 701_2024_6187_MOESM1_ESM.docx]

**Title:** Postoperative Outcomes and Electrophysiological Characteristics for Selective Dorsal Rhizotomy in Cerebral Palsy

**Author Information:**

- **Ziyad Makoshi**, MD, MSc, FRCSC; El Paso Children’s Hospital, Neurosciences Department, El Paso, Texas, USA; Texas Tech University Health Sciences Center, Department of Surgery, El Paso, Texas, USA; [zmakoshi@gmail.com](mailto:zmakoshi@gmail.com)
- **Monica Islam**, MD; The Ohio State College of Medicine, Division of Pediatric Neurology, Department of Pediatrics, Nationwide Children's Hospital, Columbus, Ohio, USA; [Monica.Islam@nationwidechildrens.org](mailto:Monica.Islam@nationwidechildrens.org)
- **Jennifer McKinney**, MD; The Ohio State College of Medicine, Division of Pediatric Neurology, Department of Pediatrics, Nationwide Children's Hospital, Columbus, Ohio, USA; [Jennifer.McKinney@nationwidechildrens.org](mailto:Jennifer.McKinney@nationwidechildrens.org)
- **Jeffrey Leonard**, MD; Nationwide Children's Hospital, Department of Pediatric Neurosurgery, Columbus, Ohio, USA; The Ohio State University Wexner Medical Center, The Ohio State University College of Medicine, Columbus, Ohio, USA; [Jeffrey.Leonard@nationwidechildrens.org](mailto:Jeffrey.Leonard@nationwidechildrens.org)

**Corresponding Author:**

Jeffrey Leonard, MD

Chief of Neurosurgery

Fellowship Director

The Robert F. & Edgar T Wolfe Foundation Endowed Chair in Neurosurgery

Nationwide Children’s Hospital

Professor of Neurological Surgery

The Ohio State College of Medicine

700 Children’s Drive

Columbus, OH 43205

Telephone 614-722-2014

**SUPPLEMENTARY TABLES**

Table 1. Frequency and percentage of preoperative compared to postoperative GMFCS level and GMFM-66 (score and percentile, n=35).

|  | **Preoperative** | | **0 – 7 m** | | **8 – 18 m** | | **≥ 19 m** | |
| --- | --- | --- | --- | --- | --- | --- | --- | --- |
| **GMFCS Level**^*^ | ***N*** | ***%*** | ***N*** | ***%*** | ***N*** | ***%*** | ***N*** | ***%*** |
| GMFCS Level (overall) | 35 | 100 |  | |  | |  | |
| Wilcoxon signed-rank test^*^ |  | | *Z*=0, *p*=1 | | *Z*=0, *p*=1 | | *Z*=-0.811, *p*=0.688 | |
| Spearman’s rho^†^ |  | | ***κ(w)=0.862 (0.737*** – ***0.988), p<0.0001*** | | ***κ(w)=0.84 (0.685*** – ***0.996), p<0.0001*** | | ***κ(w)=0.845 (0.737*** – ***0.953), p<0.001*** | |
| Ambulatory (overall)^*^ | 27 | 77.1 | 16 | 76.2 | 23 | 76.7 | 21 | 77.8 |
| Wilcoxon signed-rank test |  | | *Z*=-0.577, *p*=0.564 | | *Z*=-1, *p*=0.317 | | *Z*=0, *p*=1 | |
| Spearman’s rho |  | | ***r=0.871 (0.652*** – ***0.956), p<0.001*** | | ***r=0.88 (0.727*** – ***0.949), p<0.001*** | | ***r=0.88 (0.717*** – ***0.952), p<0.001*** | |
| 1 | 11 | 31.4 | 5 | 23.8 | 10 | 33.3 | 8 | 29.6 |
| 2 | 7 | 20 | 5 | 23.8 | 6 | 20 | 7 | 25.9 |
| 3 | 9 | 25.7 | 6 | 28.6 | 7 | 23.3 | 6 | 22.2 |
| Non-ambulatory (overall)^*^ | 8 | 22.9 | 5 | 23.8 | 7 | 23.3 | 6 | 22.2 |
| Wilcoxon signed-rank test |  | | *Z*=-1, *p*=0.317 | | *Z*=0, *p*=1 | | *Z*=-1.4, *p*=0.157 | |
| Spearman’s rho |  | | ***r=1, p<0.0005*** | | ***r=0.906 (0.807-0.956), p<0.001*** | | ***r=1, p<0.0005*** | |
| 4 | 5 | 14.3 | 4 | 19.1 | 4 | 13.3 | 5 | 18.5 |
| 5 | 3 | 8.6 | 1 | 4.8 | 3 | 10 | 1 | 3.7 |
| **GMFM-66**^‡^ | ***µE*** | ***SE*** | ***µE*** | ***SE*** | ***µE*** | ***SE*** | ***µE*** | ***SE*** |
| Score (overall) | 50.2 | 24.1 | 51.6 | 32.7 | 49.8 | 26.8 | 41.2 | 28 |
| Mean difference (Bca 95% CI), p-value | | | 1.244 (-2.395 – 4.914), p=0.464 | | 2.232 (-0.847 – 4.869), p=0.137 | | 3.663 (0.36 – 6.688), p=0.114 | |
| Ambulatory (overall) | 63.8 | 2.3 | 65 | 2.4 | 66.1 | 2.2 | 68.9 | 2.3 |
| 1 | 72.6 | 2.4 | 74.7 | 2.8 | 75.2 | 2.4 | 79.9 | 2.4 |
| 2 | 63.9 | 3 | 62.8 | 3.5 | 67.1 | 3.1 | 72.1 | 4.5 |
| 3 | 52.7 | 2.8 | 55.4 | 3.1 | 54 | 2.7 | 54.8 | 2.7 |
| Mean difference (BCa 95% CI), p-value | | | 2.45 (-1.06 – 5.441), p=0.21 | | 2.223 (-1.423 – 5.839), p=0.231 | | **5.621 (2.371** – **9.686), p=0.043** | |
| Non-ambulatory (overall) | 34.2 | 4.1 | 32.3 | 4.3 | 35.5 | 4.2 | 31.4 | 4.2 |
| Mean difference (Bca 95% CI), p-value | | | -2.144 (-6.24 – 1.417), p=0.458 | | 2.265 (-0.164 – 4.425), p=0.198 | | -3.022 (-4.975 – -0.968), p=0.098 | |
| 4 | 37.6 | 3.5 | 34.4 | 4.1 | 39.6 | 4.1 | 34.9 | 4.1 |
| 5 | 28.6 | 4.5 | 28.5 | 5 | 29.5 | 4.5 | 25.8 | 4.5 |
| Percentile (overall)^‡^ | 50.1 | 4.9 | 49.3 | 6.1 | 49.1 | 5.2 | 39.4 | 5.6 |
| Mean difference (Bca 95% CI), p-value | | | -4.6 (-15.42 – 4.933), p=0.435 | | -2.565 (-12.123 – 6.915), p=0.597 | | -11.294 (-25.591 – 2.133), p=0.096 | |
| Ambulatory (overall) | 51.6 | 5.6 | 52.0 | 6.8 | 46.6 | 5.8 | 45.6 | 6.2 |
| Mean difference (Bca 95% CI), p-value | | | -0.8 (-14.649 – 13.01), p=0.896 | | -0.7 (-16.882 – 2.688), p=0.197 | | -3.167 (-14.579 – 7.829), p=0.62 | |
| 1 | 41.7 | 8.8 | 43.4 | 10.8 | 45.1 | 9.2 | 41.9 | 9.2 |
| 2 | 61.7 | 10.8 | 55.6 | 13 | 38.4 | 11.3 | 63.9 | 15 |
| 3 | 54.4 | 9.4 | 60.1 | 11.1 | 55.1 | 9.9 | 43.9 | 9.9 |
| Non-ambulatory (overall) | 45 | 9.4 | 42.3 | 12.6 | 55.7 | 10.2 | 16.8 | 11.5 |
| Mean difference (Bca 95% CI), p-value | | | -12.2 (-39.985 – 8), p=0.352 | | 10 (-5.833 – 21.667), p=0.336 | | -38.8 (-50.8 – -11.2), p=0.145 | |
| 4 | 32 | 11.8 | 36.2 | 20.2 | 48.5 | 13.7 | 22.5 | 15.7 |
| 5 | 66.7 | 15.3 | 51.1 | 16.8 | 66.7 | 15.3 | 18.0 | 16.8 |

µE=least square means (estimate), Ambulatory=GMFCS levels 1 – 3, and non-ambulatory=levels 4 – 5.

Preoperative results were used as reference point for comparison with postoperative time points

* p-value calculated by Wilcoxon signed-rank test

† p-value calculated for weighted kappa

‡ p-value calculated by paired-sample *t*-test, reference is preoperative assessment, mean difference with bootstrap (1000 samples) with bias-corrected and accelerated (Bca) 95% CI

Table 2. Changes in mean Ashworth scores prior to and during follow up post selective dorsal rhizotomy (n=35).

| **Ashworth Score**  **Ashworth Limb** | **Preoperative** | | **0 – 3 m** | | **4 – 9 m** | | **10 – 19 m** | | **>19 months** | |
| --- | --- | --- | --- | --- | --- | --- | --- | --- | --- | --- |
|  | **µE** | **SE** | **µE** | **SE** | **µE** | **SE** | **µE** | **SE** | **µE** | **SE** |
| Ashworth Overall | 1.01 | 0.06 | 0.49 | 0.06 | 0.28 | 0.06 | 0.17 | 0.06 | 0.10 | 0.06 |
|  | *p*-value^*^ | Δµ^†^±SD | **<0.001** | 0.48±0.36 | **<0.001** | 0.74±0.99 | **<0.001** | 0.87±0.96 | **<0.001** | 0.94±1.01 |
| Upper extremity (overall) | 0.76 | 0.07 | 0.52 | 0.07 | 0.34 | 0.07 | 0.13 | 0.07 | 0.13 | 0.07 |
|  | *p*-value^*^ | Δµ^†^±SD | **0.001** | 0.13±0.63 | **<0.001** | 0.4±0.71 | **<0.001** | 0.68±0.6 | **<0.001** | 0.72±0.64 |
| Upper Extremity – Right (overall) | 0.79 | 0.08 | 0.53 | 0.07 | 0.37 | 0.08 | 0.16 | 0.08 | 0.14 | 0.08 |
|  | *p*-value^*^ | Δµ^†^±SD | **0.032** | 0.12±0.67 | **<0.001** | 0.41±0.71 | **<0.001** | 0.66±0.58 | **<0.001** | 0.74±0.67 |
| Elbow Extension | 1.07 | 0.12 | 0.60 | 0.11 | 0.59 | 0.13 | 0.35 | 0.13 | 0.36 | 0.12 |
| Elbow Flexion | 0.68 | 0.12 | 0.48 | 0.11 | 0.19 | 0.13 | 0.10 | 0.13 | 0.23 | 0.12 |
| Wrist Extension | 0.94 | 0.12 | 0.63 | 0.11 | 0.54 | 0.13 | 0.30 | 0.13 | 0.14 | 0.12 |
| Wrist Flexion | 0.61 | 0.12 | 0.45 | 0.11 | 0.19 | 0.13 | 0.00 | 0.13 | -0.03 | 0.12 |
| Finger Extension | 0.81 | 0.14 | 0.55 | 0.11 | 0.44 | 0.14 | 0.18 | 0.14 | 0.16 | 0.13 |
| Finger Flexion | 0.64 | 0.14 | 0.46 | 0.11 | 0.14 | 0.14 | 0.01 | 0.14 | -0.03 | 0.13 |
| Thumb Abduction | 0.70 | 0.14 | 0.55 | 0.11 | 0.44 | 0.14 | 0.18 | 0.14 | 0.16 | 0.13 |
| Upper Extremity – Left (overall) | 0.73 | 0.08 | 0.50 | 0.07 | 0.31 | 0.08 | 0.10 | 0.08 | 0.11 | 0.08 |
|  | *p*-value^*^ | Δµ^†^±SD | **0.012** | 0.13±0.62 | **<0.001** | 0.38±0.71 | **<0.001** | 0.7±0.62 | **<0.001** | 0.7±0.6 |
| Elbow Extension | 0.99 | 0.12 | 0.54 | 0.11 | 0.49 | 0.13 | 0.25 | 0.13 | 0.27 | 0.12 |
| Elbow Flexion | 0.73 | 0.12 | 0.48 | 0.11 | 0.24 | 0.13 | 0.15 | 0.13 | 0.14 | 0.12 |
| Wrist Extension | 0.73 | 0.12 | 0.54 | 0.11 | 0.34 | 0.13 | 0.20 | 0.13 | 0.23 | 0.12 |
| Wrist Flexion | 0.61 | 0.12 | 0.45 | 0.11 | 0.19 | 0.13 | 0 | 0.13 | -0.03 | 0.12 |
| Finger Extension | 0.70 | 0.14 | 0.52 | 0.11 | 0.38 | 0.14 | 0.07 | 0.14 | 0.11 | 0.13 |
| Finger Flexion | 0.64 | 0.14 | 0.46 | 0.11 | 0.14 | 0.14 | -0.05 | 0.14 | -0.03 | 0.13 |
| Thumb Abduction | 0.64 | 0.14 | 0.49 | 0.11 | 0.38 | 0.14 | 0.06 | 0.14 | 0.06 | 0.13 |
| Lower Extremity | 1.14 | 0.07 | 0.47 | 0.07 | 0.24 | 0.07 | 0.18 | 0.07 | 0.08 | 0.07 |
|  | *p*-value^*^ | Δµ^†^±SD | **<0.001** | 0.66±1.07 | **<0.001** | 0.9±1.06 | **<0.001** | 0.94±1.06 | **<0.001** | 1.04±1.13 |
| Hip Flexion | 0.74 | 0.1 | 0.2 | 0.057 | 0.13 | 0.042 | 0 | 0 | 0 | 0 |
| Hip Adduction | 0.61 | 0.116 | 0.17 | 0.054 | 0.12 | 0.042 | 0 | 0 | 0 | 0 |
| Knee Extension | 1.66 | 0.156 | 0.3 | 0.073 | 0.38 | 0.082 | 0.28 | 0.098 | 0.28 | 0.124 |
| Hip Extension | 0.78 | 0.101 | 0.28 | 0.076 | 0.16 | 0.046 | 0 | 0 | 0 | 0 |
| Hip Abduction | 1.32 | 0.141 | 0.19 | 0.053 | 0.19 | 0.051 | 0.14 | 0.078 | 0.17 | 0.116 |
| Knee Flexion | 0.91 | 0.116 | 0.19 | 0.053 | 0.16 | 0.046 | 0.07 | 0.048 | 0.06 | 0.039 |
| Ankle Dorsiflexion | 2.24 | 0.154 | 0.3 | 0.073 | 0.36 | 0.081 | 0.31 | 0.086 | 0.32 | 0.109 |
| Ankle plantarflexion | 0.61 | 0.093 | 0.19 | 0.053 | 0.13 | 0.042 | 0.02 | 0.017 | 0 | 0 |
| Lower Extremity – Right | 1.12 | 0.07 | 0.48 | 0.07 | 0.24 | 0.07 | 0.18 | 0.07 | 0.08 | 0.07 |
|  | *p*-value^*^ | Δµ^†^±SD | **<0.001** | 0.64±1.06 | **<0.001** | 0.88±1.07 | **<0.001** | 0.92±1.05 | **<0.001** | 1.03±1.14 |
| Hip Flexion | 0.74 | 0.11 | 0.46 | 0.1 | 0.19 | 0.12 | 0.10 | 0.11 | -0.03 | 0.11 |
| Hip Adduction | 0.69 | 0.11 | 0.46 | 0.1 | 0.19 | 0.12 | 0.09 | 0.11 | -0.03 | 0.11 |
| Knee Extension | 1.64 | 0.11 | 0.51 | 0.1 | 0.32 | 0.11 | 0.35 | 0.11 | 0.25 | 0.11 |
| Hip Extension | 0.80 | 0.11 | 0.46 | 0.11 | 0.27 | 0.12 | 0.13 | 0.11 | -0.03 | 0.11 |
| Hip Abduction | 1.33 | 0.11 | 0.49 | 0.1 | 0.21 | 0.11 | 0.17 | 0.11 | 0.12 | 0.11 |
| Knee Flexion | 0.87 | 0.11 | 0.46 | 0.1 | 0.21 | 0.11 | 0.13 | 0.11 | 0.04 | 0.11 |
| Ankle Dorsiflexion | 2.19 | 0.11 | 0.51 | 0.1 | 0.32 | 0.11 | 0.32 | 0.11 | 0.28 | 0.11 |
| Ankle plantarflexion | 0.61 | 0.11 | 0.46 | 0.1 | 0.21 | 0.11 | 0.10 | 0.11 | -0.03 | 0.11 |
| Lower Extremity – Left | 1.16 | 0.07 | 0.47 | 0.07 | 0.24 | 0.07 | 0.18 | 0.07 | 0.08 | 0.07 |
|  | *p*-value^*^ | Δµ^†^±SD | **<0.001** | 0.69±1.08 | **<0.001** | 0.93±1.06 | **<0.001** | 0.96±1.07 | **<0.001** | 1.05±1.12 |
| Hip Flexion | 0.77 | 0.11 | 0.46 | 0.1 | 0.19 | 0.12 | 0.10 | 0.11 | -0.03 | 0.11 |
| Hip Adduction | Redo | Redo | Redo | Redo | Redo | Redo | Redo | Redo | Redo | Redo |
| Knee Extension | 1.72 | 0.11 | 0.51 | 0.1 | 0.32 | 0.11 | 0.35 | 0.11 | 0.25 | 0.11 |
| Hip Extension | 0.80 | 0.11 | 0.46 | 0.1 | 0.27 | 0.12 | 0.13 | 0.11 | -0.03 | 0.11 |
| Hip Abduction | 1.94 | 0.13 | 0.28 | 0.07 | 0.25 | 0.07 | 0.33 | 0.11 | 0.29 | 0.13 |
| Knee Flexion | 0.99 | 0.11 | 0.46 | 0.1 | 0.21 | 0.11 | 0.13 | 0.11 | 0.04 | 0.11 |
| Ankle Dorsiflexion | 2.31 | 0.11 | 0.49 | 0.1 | 0.32 | 0.11 | 0.35 | 0.11 | 0.28 | 0.11 |
| Ankle plantarflexion | 0.60 | 0.10 | 0.46 | 0.1 | 0.21 | 0.11 | 0.10 | 0.11 | 0.01 | 0.11 |

* p-value calculated based on paired-sample t test

†Average difference between pre and post operative Ashworth score
